# Studies on Kinetics, Isotherms, Thermodynamics and Adsorption Mechanism of Methylene Blue by N and S Co-Doped Porous Carbon Spheres

**DOI:** 10.3390/nano11071819

**Published:** 2021-07-13

**Authors:** Yongpeng Ren, Feng Chen, Kunming Pan, Yang Zhao, Lulu Ma, Shizhong Wei

**Affiliations:** 1 School of Materials Science and Engineering, Henan University of Science and Technology, Luoyang 471003, China; REN_YP123@163.com (Y.R.); pankunming2008@haust.edu.cn (K.P.); 2 Henan Key Laboratory of High-Temperature Structural and Functional Materials, National Joint Engineering Research Center for Abrasion Control and Molding of Metal Materials, Henan University of Science and Technology, Luoyang 471003, China; gloryfire@126.com; 3 School of Environmental and Biological Engineering, Henan University of Engineering, Zhengzhou 451191, China; chenfeng871588@163.com (F.C.); malulu1001@163.com (L.M.)

**Keywords:** methylene blue, porous carbon spheres, heteroatom-doping, adsorption, mechanism

## Abstract

Heteroatom-doped carbon is widely used in the fields of adsorbents, electrode materials and catalysts due to its excellent physicochemical properties. N and S co-doped porous carbon spheres (N,S-PCSs) were synthesized using glucose and L-cysteine as carbon and heteroatom sources using a combined hydrothermal and KOH activation process. The physicochemical structures and single-factor methylene blue (MB) adsorption properties of the N,S-PCSs were then studied. The optimized N,S-PCSs-1 possessed a perfect spherical morphology with a 2–8-μm diameter and a large specific area of 1769.41 m^2^ g^−^^1^, in which the N and S contents were 2.97 at% and 0.88 at%, respectively. In the single-factor adsorption experiment for MB, the MB adsorption rate increased with an increase in carbon dosage and MB initial concentration, and the adsorption reached equilibrium within 2–3 h. The pseudo-second-order kinetic model could excellently fit the experimental data with a high R^2^ (0.9999). The Langmuir isothermal adsorption equation fitted well with the experimental results with an R^2^ value of 0.9618, and the MB maximum adsorption quantity was 909.10 mg g^−^^1^. The adsorption of MB by N,S-PCSs-1 was a spontaneous, endothermic, and random process based on the thermodynamics analyses. The adsorption mechanism mainly involved Van der Waals force adsorption, π-π stacking, hydrogen bonds and Lewis acid–base interactions.

## 1. Introduction

Dyes are widely used in textile, plastics, and the paper and pulp industries [1,2]. The characteristics of printing and dyeing wastewater, such as complex water quality, high COD value, poor biodegradability, low light permeability, and carcinogenesis and mutagenesis, lead to allergic dermatitis, skin allergy, cancer and gene mutations in the human body [3,4]. New printing and dyeing technology also aggravate wastewater treatment. Therefore, it is urgent to solve the problem of printing and dyeing wastewater treatment, as well as to provide emission standards. To date, adsorption, reverse osmosis, precipitation, biological treatment, and other decoloring technologies have been widely used to deal with printing and dyeing wastewater [4,5]. The adsorption method possesses the advantages of a high efficiency, environmental protection, simple operation, and low cost, which draws wide interest from researchers all over the world [3,6]. A new type adsorbent with a large specific surface area, high adsorption capacity, fast adsorption rate, and special surface reactivity is essential to enhance the performance of wastewater treatment.

Porous carbon has attracted broad interests because of its advantages, such as its excellent chemical and thermal stability, good mechanical stability, controllable pore structure, and high specific surface area [7,8]. In recent years, surface functionalized heteroatomic carbon materials have already been widely used as catalysts, adsorbents and as energy storage materials due to their unique physical and chemical properties. For example, Zhu’s team synthesized highly nitrogen-doped hollow carbon nanoparticles using a facile one-pot method, which showed excellent electrocatalytic activity for triiodide reduction in dye-sensitized solar cells, which was better than conventional platinum catalysts [9]. Zhang’s group demonstrated that *Pennisetum alopecuroides*-derived urea-modified activated carbon could improve the adsorption of Ni (II) from aqueous solutions because of its abundant surface nitrogen-containing functional groups [10]. Wang et al. reported that nitrogen-doped porous carbon nanosheets could lead to the effective immobilization of polysulfides and a simultaneous improvement in the reaction kinetics of sulfur species in lithium–sulfur batteries [11].

Apart from single-atom-doped carbon materials, doping with two or more kinds of heteroatoms in a carbon framework is considered to be more conducive to improving the physical and chemical properties of carbon materials, thus further broadening their application fields. Some recent studies have demonstrated this viewpoint, such as nitrogen and sulfur co-doped micro-mesoporous carbon sheets for improving Cr(VI) adsorption through synergistic effects [12], the efficient removal of methylene blue with nitrogen and oxygen co-doped three-dimensional honeycomb porous carbons [13], enhanced CO_2_ uptake and outstanding methylene blue adsorption capacities by heteroatom nitrogen and oxygen-doped porous carbon materials [14], cobalt and nitrogen co-doped porous carbon materials for enhanced capacitive deionization [15], and sulfur/nitrogen/oxygen tri-doped hierarchical porous carbon as high quality sulfur hosts in lithium–sulfur batteries [16]. Therefore, inspired by the aforementioned works, this study aimed to synthesize N and S co-doped porous carbon spheres (N,S-PCSs) derived from biomass for the treatment of printing and dyeing wastewater.

In this work, N and S co-doped porous carbon spheres (N,S-PCSs) were synthesized using glucose and L-cysteine as the carbon source and doping agent using a facile hydrothermal-KOH activation two-step method. The structure and physicochemical properties of N,S-PCSs were tested, and the adsorption properties of organic dyes in aqueous solution by N,S-PCSs were investigated in detail using methylene blue (MB) as a model dye. In addition, the adsorption kinetics, isotherms, thermodynamics, and adsorption mechanism of MB by N,S-PCSs were also discussed. This work provides an insight into sustainable biomass resources utilization and dye treatments, which could be simultaneously beneficial for emission reduction and wastewater reduction.

## 2. Materials and Methods

### 2.1. Synthesis of N,S-PCSs

Typically, for N,S-PCSs-1 preparation, 7.2 g glucose and 0.72 g L-cysteine were dissolved in 80 mL distilled water and stirred for 30 min. Then the solution was injected into the Teflon linings (100 mL) with a stainless-steel reactor. The reactor was heated at 180 °C for 12 h to finish the hydrothermal reaction, afterward, it was filtered and washed with distilled water and dried at 80 °C, the hydrothermal carbon was obtained. The hydrothermal carbon was heated in a Ni crucible under a N_2_ atmosphere at 400 °C for 1 h with a heating rate of 5 °C min^−1^ to obtain the carbonized samples. The carbonized samples and KOH were mixed at a mass ratio of 1:4, and then activated at 800 °C for 1 h under a N_2_ atmosphere with a heating rate of 5 °C min^−1^. When cooled to room temperature, the activated samples were washed with hydrochloric acid, filtered and washed with distilled water until a pH = 7 was reached, and then dried at 80 °C for 24 h. The resulting sample was marked as N,S-PCSs-1. As a contrast, the PCSs, N,S-PCSs-2 and N,S-PCSs-3 samples were also synthesized using the same method described above except with changing the L-cysteine masses to 0 g, 1.44 g and 2.88 g, respectively. Detailed information on the reagents and material characterization are shown in the Supplementary Materials (SM).

### 2.2. MB Adsorption Experiments

A total of 1 g of MB was dissolved into 1 L of distilled water to obtain the MB reserve solution (1 g L^−1^), and different concentrations of MB adsorption solutions were prepared by diluting the MB reserve solution. As with typical MB adsorption experiments, a certain mass of adsorbent was added to 50 mL of the MB solutions in conical bottles and oscillated on a thermostatic oscillator at 120 r min^−1^. After oscillation, the supernatant liquid was collected, and the concentration of residual MB solution was measured by using a UV-Vis spectrophotometer at a wavelength of 664 nm [17,18]. The standard calibration curves of the MB solutions were plotted with a high related coefficient R^2^ of 0.9993.

To evaluate the adsorption properties of different adsorbents, 4 mg of PCSs, N,S-PCSs-1, N,S-PCSs-2 and N,S-PCSs-3 were added into 5 mg L^−1^ of MB solution and oscillated at 298 K for 16 h. To confirm the optimum mass of adsorbent for removal of MB, different weights (5–15 mg) of N,S-PCSs-1 were added to 80 mg L^−1^ of MB solution and oscillated at 298 K for 16 h.

The experiments on the effect of time were carried out, ranging from 1 min to 24 h, at 298 K, containing MB with initial concentrations of 80 mg L^−1^ and 8 mg of N,S-PCSs-1, respectively. In order to explore the mechanism of the MB adsorption process, the pseudo-second-order and the internal diffusion kinetic models were used to fit the adsorption dynamics data, and the relevant equations were shown in the SM.

The initial MB concentration and temperature experiments were conducted with initial MB concentrations from 10 to 400 mg L^−1^ at 298, 308, and 318 K, respectively. The quality of N,S-PCSs-1 and contact time were set to 8 mg and 16 h, respectively. The Langmuir and Freundlich isotherm models were used to simulate the MB adsorption, and the thermodynamic parameters (ΔG^θ^, ΔH^θ^ and ΔS^θ^) were also calculated based on the isotherm data. The isotherms and thermodynamic equations are detailed in the SM.

The above-mentioned MB batch adsorption experiments were carried out as triplicate independent samplings, and all the experimental data were displayed with average values with standard deviations (less than 5%) as error bars. The distinctions between the experiment groups and the control group were tested for significance using a t-test at a significant level of 0.05.

The adsorption rate (ŋ, %) was calculated using Equation (1) (where C_0_ is the MB concentration before adsorption, mg L^−1^, C_i_ is the MB concentration after adsorption, mg L^−1^).

(1) $$\;ŋ=\frac{C_{0}\;{-\;C}_{i}}{C_{0}}\;\times \;100\%$$

The adsorption quantity (Q, mg g^−1^) was calculated using Equation (2) (where V was the volume of the MB solution, L, and m is the adsorbent quality, mg).

(2) $$Q=\frac{{V(C}_{0}\;{-\;C}_{i})}{m}\;\times \;1000$$

## 3. Results and Discussion

**Figure 1:**
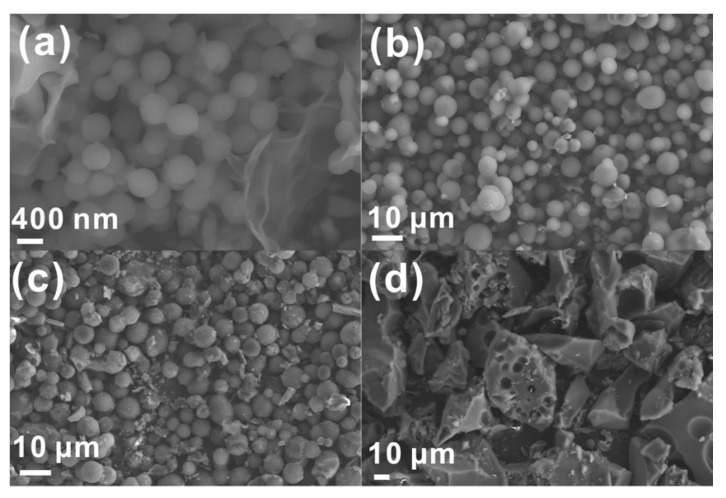
SEM images of (**a**) PCSs, (**b**) N,S-PCSs-1, (**c**) N,S-PCSs-2 and (**d**) N,S-PCSs-3.

The SEM images of PCSs, N,S-PCSs-1, N,S-PCSs-2 and N,S-PCSs-3 are shown in Figure 1. The PCS sample synthesized without L-cysteine was composed of spheres with diameters of about 300–400 nm (Figure 1a). As shown in Figure 1b, N,S-PCSs-1 was composed of smooth microspheres with diameters of about 2–8 μm, which was larger than PCS due to the addition of L-cysteine. As shown in Figure 1c, microspheres with lamellar structures on their surfaces and blocks could be discovered in the N,S-PCSs-2 sample. As a contrast, N,S-PCSs-3 with excess L-cysteine addition was composed of blocks with inverse opal structures (Figure 1d). The TEM image of N,S-PCSs-1 (Figure S1) confirmed its smooth surface and spherical morphology, corresponding to its SEM image (Figure 1b). It was demonstrated that L-cysteine addition could gradually change the morphology of the carbon material, from spheres to blocks.

Table 1 summarizes the elemental contents of N and S in N,S-PCSs-1, N,S-PCSs-2 and N,S-PCSs-3 samples according to the results of EDS analyses. Based on the elemental analyses, the N atomic percentages of the N,S-PCSs-1, N,S-PCSs-2, and N,S-PCSs-3 samples were 0.34 at%, 0.92 at%, and 1.57 at%, respectively. The S atomic percentages of the three samples were 0.4 at%, 2.75 at%, and 3.3 at%, respectively. It was demonstrated that the N and S elemental contents increased with the increasing quality of L-cysteine doping agent.

**Figure 2:**
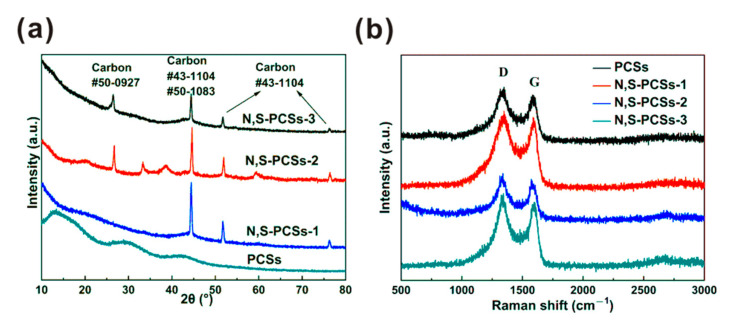
(**a**) XRD and (**b**) Raman spectra of PCSs, N,S-PCSs-1, N,S-PCSs-2 and N,S-PCSs-3.

XRD patterns of the four samples are shown in Figure 2a. The PCSs sample without N and S only showed broad peaks, corresponding to the amorphous carbon [19]. N,S-PCSs-1 with proper N and S dopant exhibited a carbon phase (JCPDS#43-1104 and #50-1083). In addition, the N,S-PCSs-2 and N,S-PCSs-3 with excess dopant exhibited additional carbon phases (JCPDS#50-0927).

Based on the Raman spectra in Figure 2b, the PCSs, N,S-PCSs-1, N,S-PCSs-2, and N,S-PCSs-3 samples exhibited two peaks at ~1580 cm^−1^ and ~1340 cm^−1^, which corresponded to the characteristic sp^2^ peak (G peak) and a disordered peak (D peak), respectively. The I_D_/I_G_ values (R value) were calculated to evaluate their graphitization degree. The R values of PCSs, N,S-PCSs-1, N,S-PCSs-2, and N,S-PCSs-3 were 1.12, 1.13, 1.15, and 1.28, respectively. The R value increased with the increase in the quality of L-cysteine (as well as increasing N/S contents), indicating the N and S dopant contributed to the decrease in the graphite structure and the increase in defects [20,21].

According to the infrared spectroscopy of N,S-PCSs-1 (Figure S2), the peak at 1105 cm^−1^ referred to S=C vibration. The peak at 1383 cm^−1^ corresponded to -COOH and C-C stretching. The peak at 1627 cm^−1^ referred to -N=N, -O-NO_2_ and -C=C stretching. The peak at 3436 cm^−1^ referred to -NH and -OH stretching [22,23]. It was demonstrated that N,S-PCSs-1 possessed the oxygen/sulfur/nitrogen-containing functional groups.

**Figure 3:**
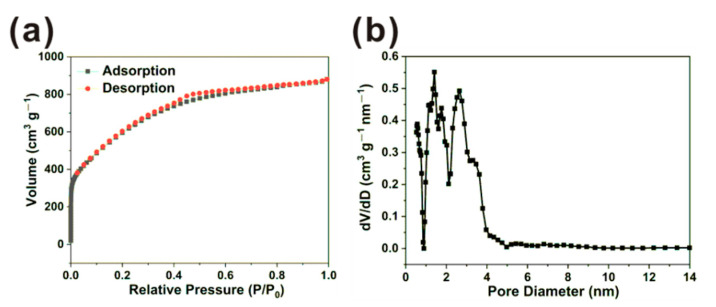
(**a**) N_2_ adsorption/desorption curves and (**b**) pore size distribution curve of N,S-PCSs-1.

The N_2_ adsorption/desorption curves and pore size distribution curve of N,S-PCSs-1 are shown in Figure 3. The adsorption/desorption curves exhibited a strong adsorption capacity under a low relative pressure, indicating the adsorption mechanism of N,S-PCSs-1 micropores and monolayers (Figure 3a). The hysteresis loop demonstrated the mesopores in N,S-PCSs-1 [24]. The pore size distribution of N,S-PCSs-1 indicated its pores were mainly distributed in micropores (diameter less than 2 nm) and mesopores between 2–4 nm (Figure 3b). As a contrast, the pores of N,S-PCSs-3 were mainly distributed in micropores (Figure S3). In addition, the specific surface area and pore volume of N,S-PCSs-1 were 1769.41 m^2^ g^−1^ and 1.23 cm^3^ g^−1^, respectively. In contrast, N,S-PCSs-3 possessed a specific surface area of ~1100 m^2^ g^−1^, which was much lower than N,S-PCSs-1. The excess L-cysteine probably led to the reduction in the specific area. Such a hierarchical micro/meso-porous structure of N,S-PCSs-1 could provide a more favorable micro-environment for transporting paths for MB, capturing more contaminants via Van der Waals forces and trapping them by physical/chemical barriers, which might endow its good adsorption properties and could be a potential candidate for contaminant remediation [25,26,27].

**Figure 4:**
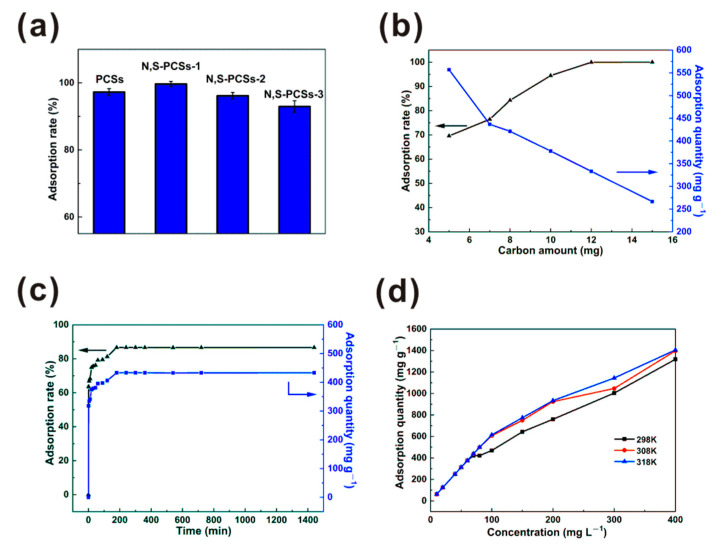
(**a**) Adsorption rates of PCSs, N,S-PCSs-1, N,S-PCSs-2, and N,S-PCSs-3: influences of (**b**) carbon amount, (**c**) contact time on adsorption rate and quantity of MB by N,S-PCSs-1, (**d**) influences of MB initial concentration and temperature on adsorption quantity of MB by N,S-PCSs-1.

Factors such as different adsorbents, carbon amount, contact time, the MB initial concentration, and temperature were used to explore the influence on MB adsorption properties. The results are shown in Figure 4. The adsorption rates of the different carbon materials were tested under the same adsorption conditions (Figure 4a). The PCSs, N,S-PCSs-1, N,S-PCSs-2, and N,S-PCSs-3 exhibited adsorption rates of 97.24%, 99.64%, 96.16%, and 92.94%, respectively. Obviously, N,S-PCSs-1 was the optimized carbon material. According to the above characterization analysis, it could be concluded that N,S-PCSs-1 had moderate N and S contents (compared to PCSs), as well as higher specific area and pore structure (compared to N,S-PCSs-2 and N,S-PCSs-3). The adsorption performance of PCSs was restricted by its low N and S. The adsorption performances of N,S-PCSs with higher N and S contents were restricted by their low specific areas and unoptimized pore structures, which might be why it has good MB adsorption performance. Hence, the following research in this work is focused on N,S-PCSs-1.

The adsorption rate and quantity with different amounts of N,S-PCSs-1 were investigated, as shown in Figure 4b. The adsorption rate increased from 69.60% to 84.27% with a carbon amount increased from 5 to 8 mg. A total of 10 mg of N,S-PCSs-1 exhibited an adsorption rate of 94.47%. The adsorption rate showed a minor growth from 99.96% to 100% when the carbon amount increased from 12 to 15 mg. With increasing N,S-PCSs-1 amounts, the adsorption rate increased by ~30%; on the other hand, adsorption quantity was reduced by ~300 mg g^−1^.

The effect of contact time on the adsorption property of MB by N,S-PCSs-1 was investigated (Figure 4c). With the adsorption time increasing from 1 to 120 min, the adsorption rate and quantity exhibited a drastic increase. The adsorption rate increased from 63.58% to 81.27%, and the adsorption quantity increased from 317.88 to 406.33 mg g^−1^. With the adsorption time increasing from 120 to 180 min, the adsorption rate and quantity exhibited a minor increase. The adsorption rate increased from 81.27% to 86.61%, and the adsorption quantity increased from 406.33 to 433.03 mg g^−1^. With the adsorption time exceeding 180 min, the adsorption rate was maintained at ~86%, and the adsorption quantity was maintained at 433 mg g^−1^; hence, the adsorption equilibrium time of MB by N,S-PCSs-1 could be 180 min.

The influences of MB initial concentration and temperature on the adsorption quantity of MB by N,S-PCSs-1 were investigated, as shown in Figure 4d. At a temperature of 298 K, with the initial MB concentration being less than 60 mg L^−1^, the adsorption quantity increased from 62.5 to 375 mg g^−1^. With the initial MB concentration increasing from 70 to 400 mg L^−1^, the adsorption quantity increased from 420.9 to 1318.5 mg g^−1^. With the initial MB concentration increasing to 400 mg L^−1^, the adsorption quantity approach its highest value. This phenomenon might be attributed to the hierarchical porous structures, because the MB adsorption of the micro-pores was faster than that of the meso-pores. From Figure 4d, we can also see that the adsorption quantity increased with an increase in temperature. The adsorption quantity exhibited a significant increase at 298 to 308 K, nevertheless, there was a minor increase at 308 to 318 K, demonstrating that MB absorption by N,S-PCSs-1 was an endothermic process [28,29].

**Figure 5:**
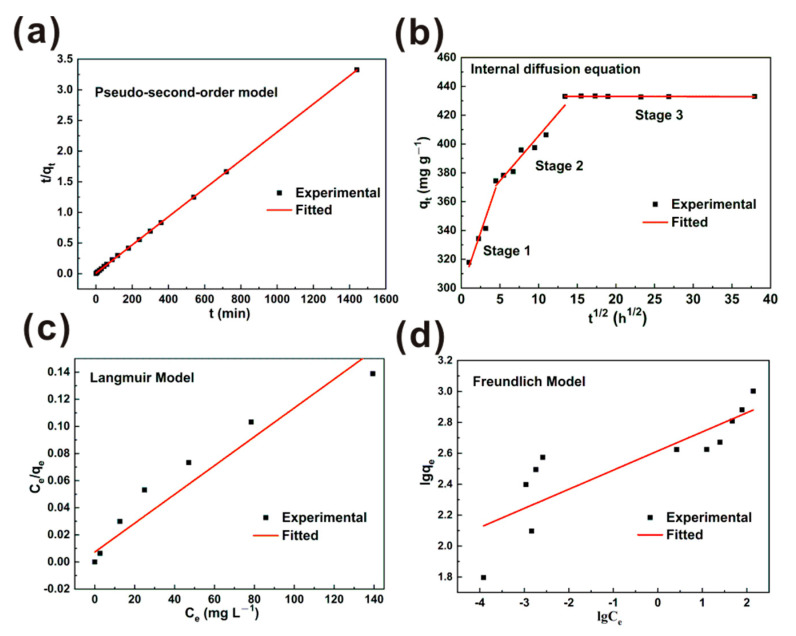
Experimental and fitted data of adsorption kinetics based on (**a**) pseudo-second-order model and (**b**) internal diffusion model; experimental and fitted data of adsorption isotherms based on the (**c**) Langmuir model and (**d**) the Freundlich model.

Based on time-dependent adsorption data (see Figure 4c), the pseudo-second-order and internal diffusion kinetics models were used to simulate MB adsorption by N,S-PCSs-1, the results are shown in Figure 5a,b and Table 2. Based on the pseudo-second-order model (Figure 5a), the calculated q_e_ was 434.78 mg g^−1^, which was well in accordance with the experimental value q_exp_ (433.01 mg g^−1^). The calculated K_2_ was 0.00067, and the R^2^ was 0.9999. This manifested in the adsorption kinetics of MB by N,S-PCSs-1 being well described by the pseudo-second-order model, and the adsorption process was chemisorptions with all kinds of interactions, such as H-bond formations, electrostatic attractions, Van der Waals forces, etc. [27,30]. Based on the internal diffusion model, the adsorption process was divided into three stages (Figure 5b). Stage 1, 2 and 3 corresponded to the fast physisorption by micro-pores, the chemisorption by the functional groups on the surfaces of the N,S-PCSs-1 and the slow adsorption near equilibrium, respectively [12,31]. For all the tested MB concentrations, the lines of the three stages did not go through the origin of coordinates, suggesting that internal diffusion was not the sole rate-controlling step [31].

The Langmuir and Freundlich isotherm models were used to simulate the MB adsorption by N,S-PCSs-1, and the results are shown in Figure 5c,d and Table 2. The R^2^ of the Langmuir model was 0.9410, which was larger than that of the Freundlich model (0.7720), indicating that the MB adsorption by N,S-PCSs-1 could be well described by the Langmuir model [28,32]. This also demonstrated that MB molecules formed a homogenous monolayer coverage on the surface of N,S-PCSs-1 [33]. Based on the Langmuir model, the maximum adsorption quantity of MB by N,S-PCSs-1 was 909.10 mg g^−1^. According to the Freundlich model, 1/n was 0.12, indicating a good affinity between MB and N,S-PCSs-1 [31].

Based on adsorption experiments at different temperatures (Figure 4d), ΔG^θ^ and K_d_ were obtained. A fitted linear relation between lnK_d_ and 1/T was devoted to the calculation of ΔH^θ^ and ΔS^θ^ (Figure S4). The results are shown in Table 3. The values of ΔG^θ^ at 298, 308 and 318 K were less than 0, indicating that MB adsorption was a spontaneous process [34]. The absolute values of ΔG^θ^ increased with increasing temperature, indicating that the increase in temperature was a benefit to MB adsorption. The value of ΔH^θ^ was higher than 0, indicating the MB adsorption was an endothermal process [35]. The value of ΔS^θ^ was larger than 0, suggesting the MB adsorption by N,S-PCSs-1 was a randomness process [36].

**Figure 6:**
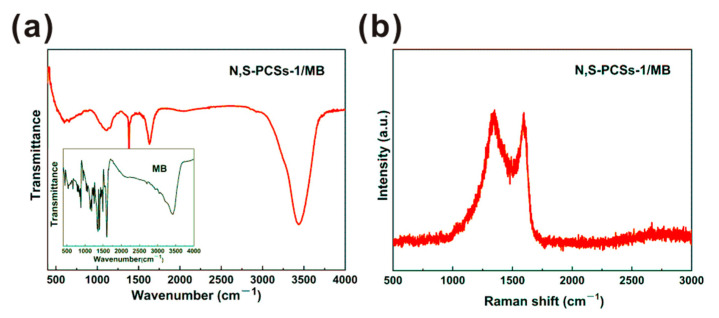
Infrared spectroscopy of (**a**) N,S-PCSs-1/MB and (**a**) inset MB and (**b**) Raman spectrum of N,S-PCSs-1/MB.

N,S-PCSs-1 after MB adsorption (N,S-PCSs-1/MB) showed a minor morphology change, indicating its structural stability, which also showed that the adsorption of pores via Van der Waals forces played an important role in MB adsorption (Figure S5) [25]. The infrared spectroscopy and Raman spectroscopy of N,S-PCSs-1 after MB adsorption are shown in Figure 6. The infrared spectroscopy indicated that all of the peaks have blue shift, such as the S=C and C-C peak shifted from 1105 cm^−1^ and 1112 cm^−1^ to 1383 cm^−1^ and 1385 cm^−1^, respectively (Figure 6a). The blue shift could be attributed to the stress change caused by MB adsorption. In addition, compared to other peaks, the intensity of C-C peak exhibited an extra increase, which was attributed to the extra C-C bond introduced by the adsorbed MB molecules. Moreover, the peaks of -NH and -OH for N,S-PCSs-1 also migrated after MB adsorption, which suggested that the hydrogen bond could be involved in MB adsorption [31]. Infrared spectrum analysis showed that the surface functional groups of N,S-PCSs-1 participated in the adsorption of MB, which was consistent with the results of kinetics [28,31]. The Raman spectrum indicated that the I_D_/I_G_ of N,S-PCSs-1/MB was 1.09, which was lower than that of N,S-PCSs-1 (1.13). This phenomenon could be attributed to the increased sp^3^ carbon atom introduced by MB (Figure 6b).

**Figure 7:**
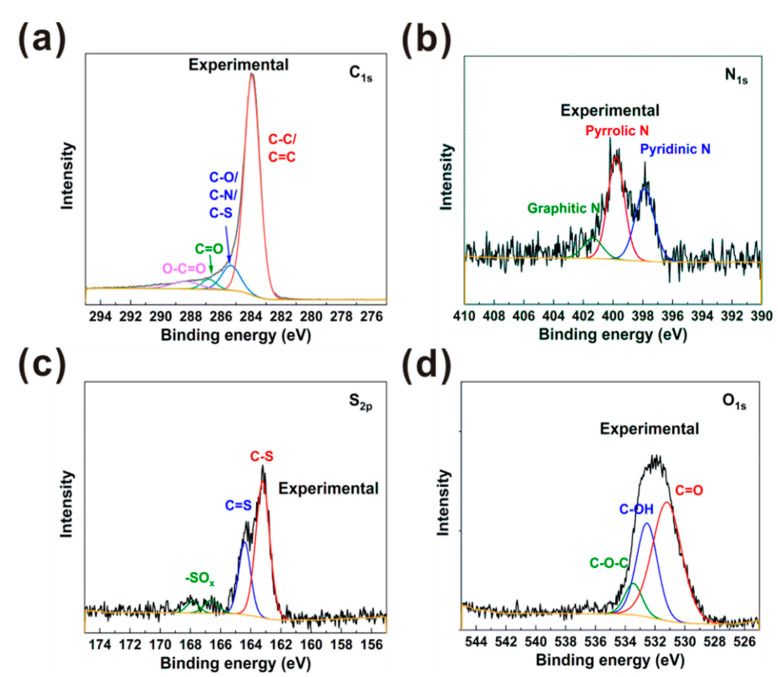
N,S-PCSs-1 XPS spectrums of (**a**) C_1s_ (**b**) N_1s_, (**c**) S_2p_ and (**d**) O_1s_.

In order to further probe the adsorption mechanism of MB by N,S-PCSs-1, XPS tests were carried out. The XPS spectra of N,S-PCSs-1 were shown in Figure 7. According to XPS, the C, O, N and S contents of N,S-PCSs-1 were 89.45 at%, 6.7 at%, 2.97 at%, and 0.88 at%, respectively. The C_1s_ spectrum with a binding energy of ~284 eV was deconvoluted into 4 peaks centered at 283.9 eV, 285.3 eV, 286.8 eV, and 288.4 eV, corresponding to C=C/C–C, C–O/C–N/C–S, C=O, and O–C=O, respectively [31,37]. The N_1s_ spectrum at a binding energy of 398–400 eV was deconvoluted into 3 peaks centered at 397.9 eV, 399.8 eV, and 401.4 eV, corresponding to pyridinic N, pyrrolic N, and graphitic N, respectively [24,37]. The S_2p_ spectrum at a binding energy of ~163 eV was deconvoluted into 3 peaks centered at 163.2 eV, 164.4 eV, and 166.7~168.0 eV, corresponding to C–S, C=S, and -SO_x_, respectively [12,38]. The O_1s_ spectrum at a binding energy of ~532 eV was deconvoluted into 3 peaks centered at 531.2 eV, 532.5 eV, and 533.5 eV, corresponding to C=O, C–OH, and C–O–C, respectively [13,39]. According to the above XPS analysis, we can see that N,S-PCSs-1 had a π-π structure in its skeleton and the oxygen/sulfur/nitrogen-containing functional groups on the surface, simultaneously, MB possessed aromatic rings in their structures. Therefore, a π-π stacking interaction should be formed between N,S-PCSs-1 and MB [13]. In addition, the MB structure contained organic ammonium ions, and the N^+^ position had the capacity to receive electrons (acting as a Lewis acid), while the N, S and O atoms of N,S-PCSs-1 contained lone pair electrons, which could act as a Lewis base to donate electrons. Thus, the Lewis acid–base interactions between N,S-PCSs-1 and MB could also be beneficial to MB adsorption [12,13,40].

**Figure 8:**
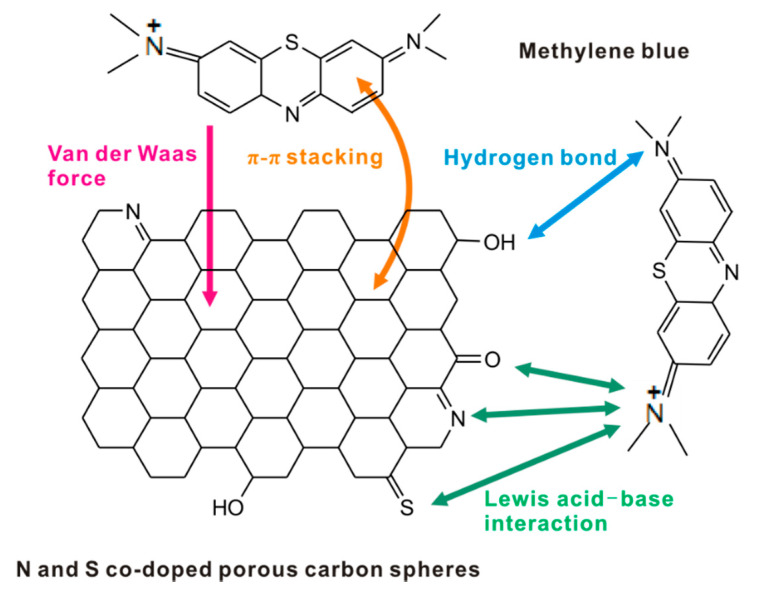
Schematic illustration of the MB adsorption mechanism by N,S-PCSs-1.

Therefore, according to the results of the above analysis, the MB adsorption mechanism by N,S-PCSs-1 were proposed to the Van der Waals force adsorption, π-π stacking, hydrogen bond and Lewis acid–base interaction, as shown in Figure 8.

## 4. Conclusions

In conclusion, a facile method was utilized to prepared N and S co-doped porous carbon spheres (N,S-PCSs) using glucose and L-cysteine as the carbon precursor and heteroatom source. The as-prepared carbon material possessed good physical and chemical properties and was able to exhibit good properties for MB adsorption. The MB adsorption onto the optimized N,S-PCSs-1 material was fitted to follow the pseudo-second-order kinetic model and Langmuir isotherm model. The MB adsorption by N,S-PCSs-1 was a monolayer chemisorption, and the maximum adsorption quantity of MB was as high as 909.10 mg g^−1^. Thermodynamics calculation showed that the MB adsorption was a spontaneous, endothermic, and random process. The mechanism for MB adsorption onto N,S-PCSs-1 was proposed as the Van der Waals force adsorption, π-π stacking, hydrogen bond and Lewis acid–base interactions. We believe that N,S-PCSs-1 is a green and sustainable adsorbent with good removal efficiency for the treatment of printing and dyeing wastewater.

## Figures and Tables

**Table 1:** Elemental contents of N,S-PCSs-1, N,S-PCSs-2 and N,S-PCSs-3.

Samples	Type	Elemental Contents	
		N	S
N,S-PCSs-1	Weight percentage	0.39	1.05
	(Atomic percentage)	−0.34	−0.4
N,S-PCSs-2	Weight percentage	0.98	6.7
	(Atomic percentage)	−0.92	−2.75
N,S-PCSs-3	Weight percentage	1.71	8.24
	(Atomic percentage)	−1.57	−3.3

**Table 2:** Fitted data of adsorption kinetics and isotherms.

	(Model) Equation	Fitted Parameter		Fitted Equation
Adsorption kinetics	(Pseudo-second-order) $\frac{t}{q_{t}}=1/k_{2}q_{e}^{2}+t/q_{e}$ x = t, y = t/q_t_	k_2_	0.00067	y = 0.0023x + 0.0079
		q_e_ (mg g^−1^)	434.78	
		q_exp_ (mg g^−1^)	433.01	
		R^2^	0.9999	
	(Internal diffusion) qt=kpt12+C x = t^1/2^, y = q_t_	(Stage 1) k_p_	15.76	y = 15.756x + 299.18
		(Stage 1) R^2^	0.9469	
		(Stage 2) k_p_	6.25	y = 6.2491x + 343.16
		(Stage 2) R^2^	0.9446	
		(Stage 3) k_p_	−0.011	y = −0.0106x + 433.24
		(Stage 3) R^2^	0.9786	
Adsorption isotherms	(Langmuir model) $\frac{C_{e}}{q_{e}}=\frac{1}{{\mathrm{bq}}_{m}}+\frac{C_{e}}{q_{m}}$ x = C_e_ y = C_e_/q_e_	qm (mg g^−1^)	909.10	y = 0.0011x + 0.0073
		b	0.15	
		R^2^	0.9410	
	(Freundlich model) ${\mathrm{lgq}}_{e}=\mathrm{lg}\;k+\frac{1}{n}{\mathrm{lgC}}_{e}$ x = lg C_e_, y = lg q_e_	k	411.81	y = 0.1237x + 2.6147
		1/n	0.12	
		R^2^	0.7720	

**Table 3:** ΔG^θ^, ΔH^θ^ and ΔS^θ^ data of MB adsorption by N,S-PCSs-1.

ΔH^θ^ (kJ mol^−1^)	ΔS^θ^ (kJ mol^−1^ k^−1^)	ΔG^θ^ (kJ mol^−1^)		
		298 K	308 K	318 K
5.57	19.76	−0.27	−0.61	−0.66

## Data Availability

Not applicable.

## Supplementary Materials

The following are available online at https://www.mdpi.com/article/10.3390/nano11071819/s1, Figure S1: TEM image of N,S-PCSS-1, Figure S2: Infrared spectroscopy of N,S-PCSs-1, Figure S3: N2 adsorption/desorption curves and pore size distribution curve of N,S-PCSs-3, Figure S4: Adsorption thermodynamics of MB by N,S-PCSs-1, Figure S5: SEM image of N,S-PCSs-1/MB, Experimental Details.
