# Supplementary material for: Studies on Kinetics, Isotherms, Thermodynamics and Adsorption Mechanism of Methylene Blue by N and S Co-Doped Porous Carbon Spheres
Source: Nanomaterials (Basel). 2021 Jul 13;11(7):1819. doi: 10.3390/nano11071819 (PMC8308386; doi:10.3390/nano11071819)
Supplement: Supplementary file 1 [file nanomaterials-11-01819-s001.zip › nanomaterials-1269663-supplementary.pdf]

nanomaterials

MDPI

Supplementary Materials

Studies on Kinetics, Isotherms, Thermodynamics and Adsorption Mechanism of Methylene Blue by N and S Co-Doped Porous Carbon Spheres

Yongpeng Ren [1,2], Feng Chen [3], Kunming Pan [1,2], Yang Zhao [2], Lulu Ma [3] and Shizhong Wei [1,2,*]

[1] School of Materials Science and Engineering, Henan University of Science and Technology, Luoyang 471003, China; REN_YP123@163.com (Y.R.); pankunming2008@haust.edu.cn (.K.P)
[2] Henan Key Laboratory of High-Temperature Structural and Functional Materials, National Joint Engineering Research Center for Abrasion Control and Molding of Metal Materials, Henan University of Science and Technology, Luoyang 471003, China; gloryfire@126.com
[3] School of Environmental and Biological Engineering, Henan University of Engineering, Zhengzhou 451191, China; chenfeng871588@163.com (F.C.); malulu1001@163.com (L.M.)
* Correspondence: hnwsz@126.com

Experimental Details

1. Materials and Reagents

Glucose, potassium hydroxide and absolute ethyl alcohol were purchased from Tianjin Fengchuan chemical reagent technology Co., Ltd, Tianjin, China. L-cysteine was obtained from Tianjin Xianshuigu industrial park, Tianjin, China. Methylene blue was purchased from Shanghai reagent Co., Ltd, Shanghai, China. Hydrochloric acid was purchased from Luoyang Haohua chemical reagent Co., Ltd, Luoyang, China. The reagents in this work were used without further purification. The solutions were prepared with distilled water all the experiments.

2. Characterization

The morphologies and structures of the samples were analyzed by scanning electron microscope (SEM, Quanta250, FEI, Hillsboro, USA) with an energy-dispersive spectrometer (EDS) and transmission electron microscope (TEM, JEM2100, JEOL, Tokyo, Japan). For SEM, the maximum pressure of the sample chamber was 2600Pa, and the accelerating voltage was 200V-30kV. The maximum acceleration voltage of TEM was 200kV. The surface functional groups of the samples were tested by fourier transform infrared spectrum (FTIR, Nicolet 6700, Thermo Fisher, Waltham, USA), and the spectrum range was 4000-350 cm^{-1}. The X-ray diffraction (XRD, D8 ADVANCE, Bruker) was performed at a scan rate of 10°/min to analyze the phase structure. The molecular and crystal structure was characterized by micro laser Raman spectroscopy (Raman, inVia Reflex, Renishaw, London, UK). The specific surface area, pore volume and pore size distributions of the carbon samples were calculated by using an automatic specific surface and porosity analyzer (Autosorb iQ 2 MP-XR, Quantachrome, Boynton Beach, USA).

3. Kinetics, Isotherms, Thermodynamics Equations

3.1. Kinetics Models of Adsorption

Pseudo-Second-Order Equation

The pseudo-second-order equation was shown as the equation (1).

$$\frac{t}{q_t} = 1/k_2\, q_e^2 + t/q_e \quad (1)$$

Where, t was adsorption time, q_e was the equilibrium adsorption quantity, q_t was the adsorption quantity at time t, and k_2 was the second-order reaction rate constant.

3.2. Internal Diffusion Equation

The internal diffusion equation was shown as the Equation (2).

$$q_t = k_p t^{1/2} + C \quad (2)$$

Where, k_p was the internal diffusion rate constant.

3.3. Isotherms Models of Adsorption

3.3.1. Lamgmuir Model

The Lamgmuir model could be shown as Equation (3).

$$\frac{C_e}{q_e} = \frac{1}{bq_m} + \frac{C_e}{q_m} \quad (3)$$

Where, q_m was the maximum adsorption quantity, C_e was the equilibrium concentration of the adsorbate, and b was the Lamgmuir adsorption equilibrium constant.

3.3.2. Fruenlich Model

The Fruenlich model was shown in Equation (4).

$$\lg q_e = \lg k + \frac{1}{n}\lg C_e \quad (4)$$

Where, k and n were adsorption constants. The 1/n value of 0–1 indicated the easy adsorption processes, nevertheless, the 1/n larger than 2 indicated the difficult adsorption processes.

3.4. Thermodynamics of Adsorption

The thermodynamics parameters of adsorption could be calculated via the equations as follows.

$$\Delta G^{\theta} = -RT\ln K_d \quad (5)$$

$$K_d = \frac{mq_e}{C_e V} \quad (6)$$

$$\Delta G^{\theta} = \Delta H^{\theta} - T\Delta S^{\theta} \quad (7)$$

Where, ΔG^{θ} was the Gibbs free energy change, R was the gas constant (8.314J·mol^{-1}·K^{-1}), K_d was the equilibrium constant, m was the weight of the adsorbent, V was the volume of the solution, ΔH^{θ} was the adsorption enthalpy change, ΔS^{θ} was the adsorption entropy change.

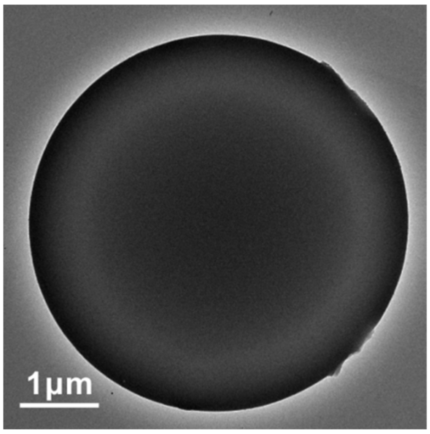

Figure S1. TEM image of N,S-PCSS-1.

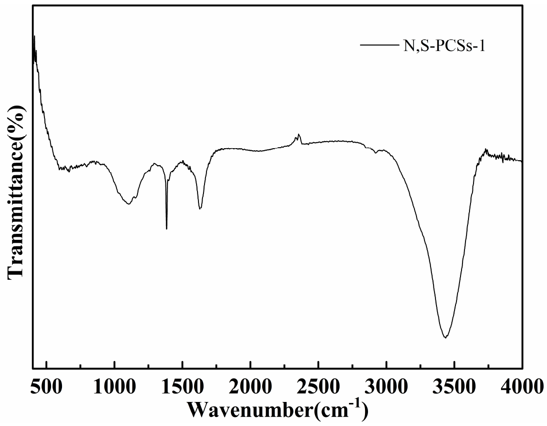

Figure S2. Infrared spectroscopy of N,S-PCSs-1.

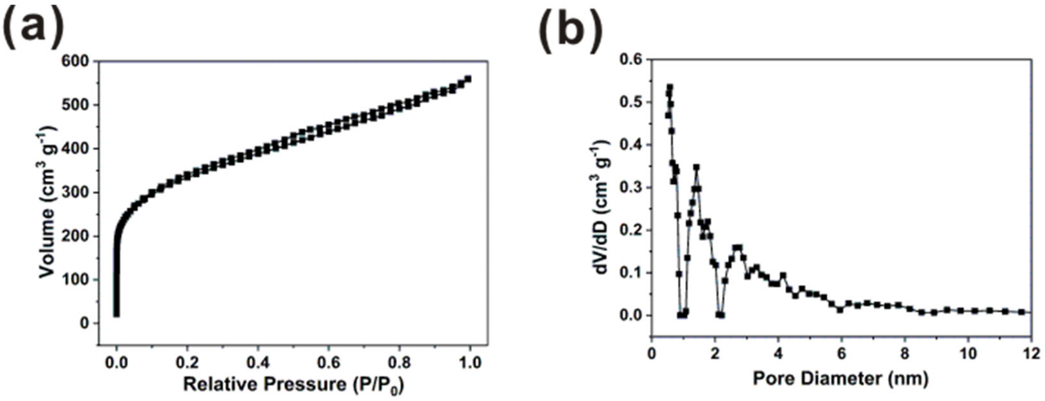

Figure S3. (**a**) N_2 adsorption/desorption curves and (**b**) pore size distribution curve of N,S-PCSs-3.

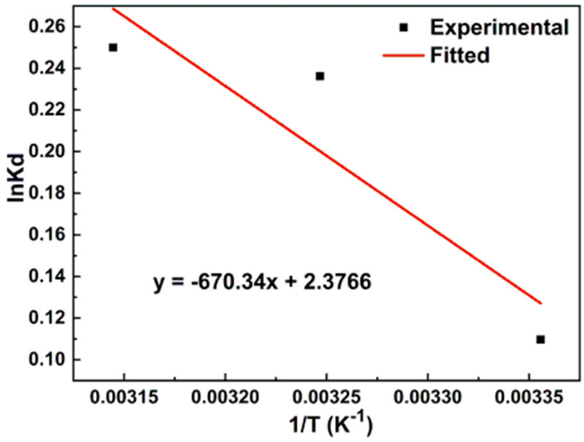

Figure S4. Adsorption thermodynamics of MB by N,S-PCSs-1.

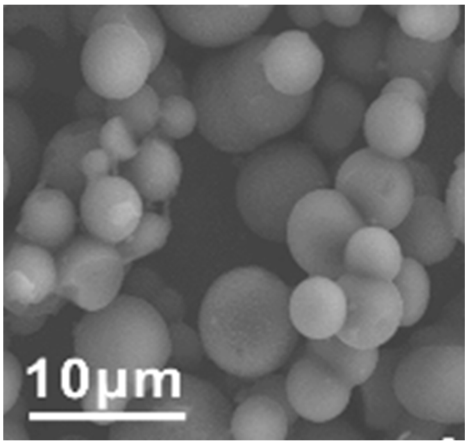

Figure S5. SEM image of N,S-PCSs-1/MB.
